# Safe Handover or Lost in Transition?—A Retrospective Evaluation of Pharmacist Referrals to Primary Care Following Hospital Discharge

**DOI:** 10.3390/pharmacy14050122

**Published:** 2026-08-20

**Authors:** Katarina Wickman, Cecilia Lenander, Gabriella Caleres, Sara Modig

**Affiliations:** 1 Center for Primary Health Care Research, Department of Clinical Sciences Malmö, Lund University, 202 13 Malmö, Sweden; cecilia.lenander@med.lu.se (C.L.); gabriella.caleres@med.lu.se (G.C.); sara.modig@med.lu.se (S.M.); 2 University Clinic Primary Care Skåne, 223 81 Lund, Sweden; 3 Department of Medicines Management and Informatics in Skåne County, 291 89 Kristianstad, Sweden

**Keywords:** interprofessional relations, medication-related problem, medication review, medication safety, patient discharge, pharmacist, primary care, referral, transition of care

## Abstract

The medication-related problems identified by clinical pharmacists during hospitalisation may be better addressed after patient discharge and recovery from acute illness. This approach enables follow-up and may reduce the risks associated with multiple medication changes. However, inadequate information transfer at discharge is a recognised patient safety risk. Referral-based communication between clinical pharmacists and general practitioners (GPs) is a novel procedure in southern Sweden. This retrospective observational study aimed to describe the patient population, medication-related problems, their clinical relevance, and resulting GP actions. Data were collected from pharmacist referrals and electronic medical records between Q4 2021 and Q4 2023. Medication-related problems were categorised, recommendations were assessed for clinical relevance, and follow-up within six months after discharge was analysed to determine whether GPs accepted the recommendations. Forty patients with 58 medication-related problems were included. Women accounted for 70% of the patients, the median age was 79.5 years, and 75% had extensive polypharmacy. Adherence problems were most common (22%). Overall, 98% of the recommendations were considered clinically relevant. Of the 48 assessable recommendations, 77% were implemented in primary care. No statistical association between clinical relevance and implementation was detected (odds ratio 2.27, *p* = 0.247, CI 0.57–9.04). Pharmacist referrals at discharge may support information transfer and provide clinically relevant recommendations that improve medication safety.

## 1. Introduction

Older persons with polypharmacy are at increased risk of medication-related problems (MRPs) due to age-related physiological changes [1,2]. MRPs may, for example, include inappropriate dosing in relation to renal function, adverse drug reactions, and clinically significant drug–drug interactions [2]. Clinical pharmacists frequently identify MRPs in patients that have been hospitalised. However, some MRPs are more appropriately managed in primary healthcare after recovery from acute illness [3], thus highlighting the need for effective communication between healthcare providers. Inadequate information transfer at hospital discharge is a recognised patient safety risk [4,5], and referrals have been proposed as a strategy to improve continuity of care during healthcare transitions [5]. The importance of safe information transfer has gained increasing attention in recent years [5,6,7]. The Swedish healthcare system is structured such that patients are typically registered with a general practitioner (GP) for primary care, who serves as the central coordinator of their healthcare. The GP is often responsible for managing multiple chronic conditions and plays a key role in ensuring continuity and coordination of care. Clinical pharmacist support remains limited in both hospital and primary care settings. In addition, community pharmacies lack formal communication interfaces with the healthcare system, beyond the transmission of prescriptions. This organisational structure limits their ability to support healthcare professionals to the same extent as in countries such as the United Kingdom (UK). In the UK, the Discharge Medicines Service (DMS) has been implemented, through which referrals from hospitals are sent to community pharmacies to improve medication continuity after discharge, enhance communication regarding medication changes between secondary and primary care, and reduce avoidable hospital readmissions [8]. A study estimated that the DMS had reduced the risk of readmissions [9]. Studies from Denmark and the Netherlands report that pharmacist involvement in the discharge procedure could improve the quality of medicine-related healthcare services [10], and 88% of GPs expressed that pharmacotherapeutic advice from clinical pharmacists was valued [11]. Studies evaluating pharmaceutical interventions at discharge have reported mixed results for hospital readmissions but consistent reductions in MRPs [12,13,14]. In several Swedish counties, referrals regarding MRPs from hospital clinical pharmacists to subsequent care providers have recently been introduced. Clinical pharmacists recognise their role in promoting patient safety and the safe transfer of medication-related information [15]. In addition, discharge information from hospital physicians is often incomplete, while inadequate referral requests are common [16,17]. Discharge information regarding medication-related information is also perceived as more reliable by GPs when interprofessional medication reviews have been performed during hospitalisation [4,16].

Evidence regarding pharmacist-initiated referrals in Sweden remains limited, and international findings are heterogeneous [11,18,19,20]. However, qualitative studies from the UK have described positive collaboration between pharmacists and GPs [15,21]. As healthcare systems differ between countries, further evaluation in the Swedish context is warranted.

The aims of this study were to describe the patient population and categorise identified MRPs, assess the clinical relevance of recommendations included in the referrals initiated by hospital clinical pharmacists, and examine the actions taken by GPs after receiving the referrals.

## 2. Materials and Methods

### 2.1. Study Design, Setting, and Participants

This study was a retrospective cross-sectional observation study based on data derived from referrals initiated by clinical pharmacists at discharge from all eight public hospitals in Skåne County, southern Sweden. A rough estimate indicated that the hospitals employed approximately 30 pharmacists. The referral process was introduced in 2020 but had not yet been fully implemented during the study period. Additional data were collected from electronic medical records (EMRs) from public and private primary care centres and hospitals. Patients were excluded if they were younger than 18 years of age, had a protected identity, or were unable to provide informed consent.

### 2.2. Procedure

Before study initiation, all clinical pharmacists received updated information regarding the referral process, associated regulations, and practical procedures. Medication reconciliations and medication reviews were conducted according to a well-established clinical pharmacy model used in the region for approximately 20 years [22]. Identified MRPs and corresponding recommendations were discussed with the responsible hospital physician. MRPs considered more appropriate for management after discharge were eligible for referral to primary care according to the new procedure. The decision to initiate a referral was based on clinical judgement, without predefined criteria. Inclusion criteria for the referral process and study participation included the patient’s ability to provide informed consent and provision of informed consent. Patients who declined study participation continued to receive standard care. The pharmacist and physician jointly determined who was most suitable to prepare the referral. For example, if several medical issues apart from MRPs needed to be addressed or if the pharmacist was unavailable at discharge, the physician could prepare the referral instead. Referrals were documented as free text in the EMR and transferred together with the discharge summary and medication list to the primary care physician. Referrals were monitored by the referring hospital unit until receipt by primary care was confirmed. In routine practice, referrals to primary care are assessed and prioritised by the receiving primary care physician.

### 2.3. Data Collection

Data were collected between Q4 2021 and Q4 2023. Patient characteristics, including age, sex, diagnoses, renal function, ward type, discharge medications, and multidose drug dispensing [23], were retrieved from the EMRs. Information from referrals was used to categorise MRPs, involved medication groups according to the Anatomical Therapeutic Chemical classification system, ATC [24], and the clinical relevance of recommendations. Follow-up data regarding referred MRPs were collected from primary care EMRs and categorised as *solved*, *partly resolved*, *not resolved*, or *could not be assessed* within six months after discharge. The category *solved* included acceptance of recommendations that could be detected as notes in the EMR, new prescriptions or medication discontinuation, laboratory monitoring in accordance with recommendation, or patient information. The category *partly resolved* involved actions not entirely taken in accordance with recommendations, such as laboratory monitoring due to adverse reaction or interactions instead of medication discontinuation or other actions that could prevent a potential MRP in the future. Recommendations declined or acknowledged but not implemented by the GP, as well as cases with a closed EMR following the patient’s death, were classified as *not resolved*. The category *could not be assessed* included cases in which no comment was documented in the referral response in the EMR and where relevant information could not be obtained from other sources due to restricted access, such as the medication list for the multidose dispensing system. In addition, actions were categorised according to time to implementation: within 1 week, 1–4 weeks, 1–3 months, or 3–6 months. The six-month follow-up period was chosen to allow sufficient time for assessment and follow-up in primary care while maintaining relevance to the referral. MRPs were categorised according to Strand et al. by one pharmacist (KW) and cross-checked by a geriatrician and a GP [25]. Clinical relevance of pharmacist recommendations was independently assessed according to Hatoum et al. by the same geriatrician and GP [26]. Discrepancies were resolved through consensus discussion. Cases with insufficient information in the referral were noted. The Hatoum scale was selected because it is a well-established tool for evaluating the clinical relevance of pharmacists’ recommendations and has previously been applied in both hospital and primary care research. GP actions based on referral recommendations were assessed from EMR documentation, including clinical notes, medication lists, and prescriptions, and categorised by one pharmacist (KW). A random sample, which was generated using Microsoft Excel 365, was cross-checked by one GP. In two inconclusive cases, consensus was reached within the full research group.

### 2.4. Data Analysis

Descriptive statistics are used to summarise patient characteristics, number of medications, multidose drug dispensing, medication groups, MRPs, clinical relevance assessments and GPs’ acceptance of recommendations. Continuous variables with skewed distributions are presented as median and interquartile range. Inter-rater agreement was evaluated using weighted Cohen’s kappa [27]. Binary logistic regression analysis was performed to examine the association between GPs’ acceptance of recommendations (i.e., solved and partly solved) and the degree of clinical relevance (Hatoum ranking). Fisher’s exact test was used to analyse differences in adherence-related MRPs between patients with and without multidose drug dispensing. Statistical analyses were performed using IBM SPSS Statistics version 31 for descriptive analyses of study characteristics, calculation of weighted Cohen’s kappa, and binary logistic regression analysis.

### 2.5. Ethics

This study was conducted in accordance with the Declaration of Helsinki—Ethical Principles for Medical Research Involving Human Participants [28] and was approved by the Swedish Ethical Review Authority (registration number 2021-02588). All patients provided written informed consent to participate.

## 3. Results

### 3.1. Patient Characteristics

**Figure 1:**
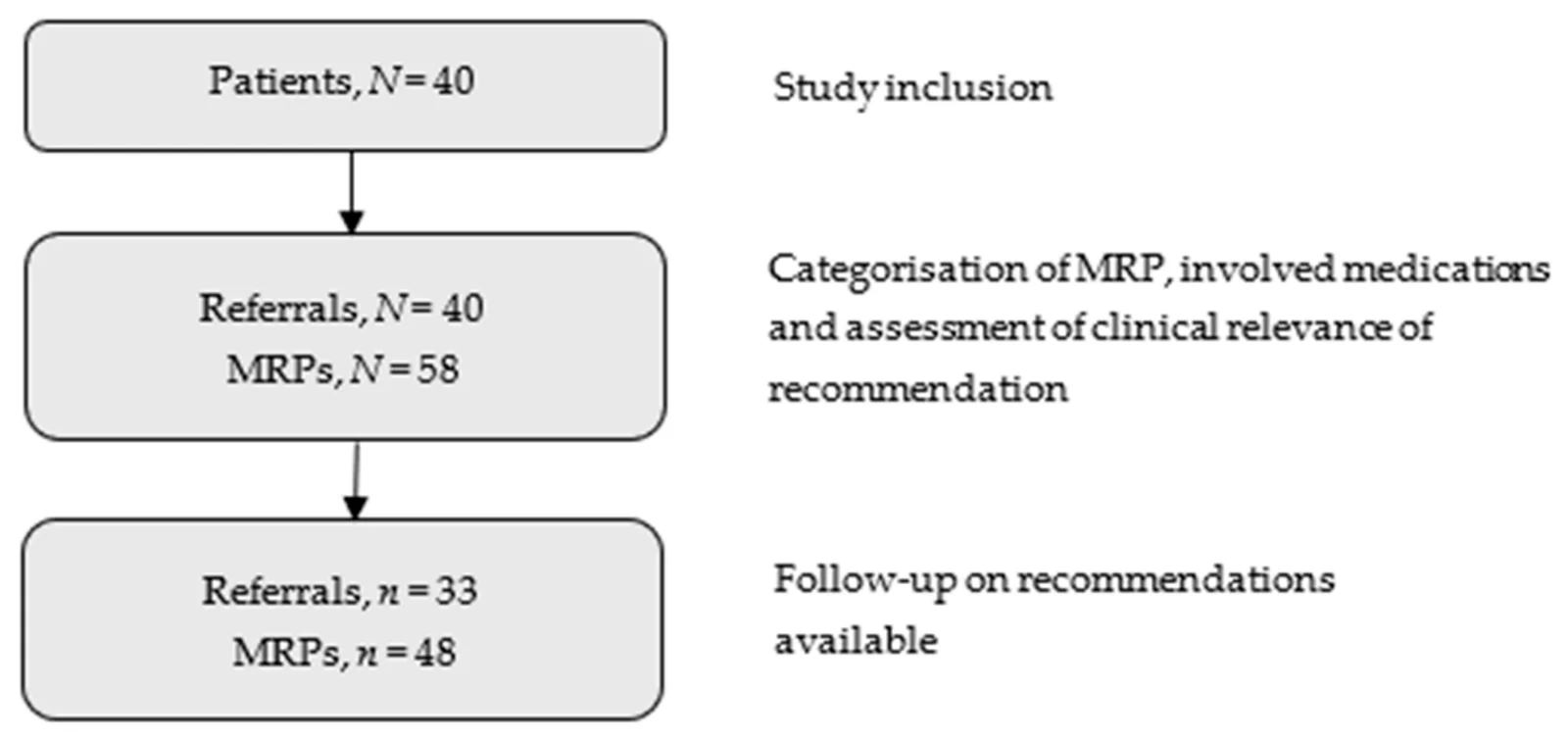
Flowchart of patients included in this study.

A total of 40 patients were included in this study, with pharmacist-initiated referrals generated according to the new procedure (Figure 1). Women accounted for 70% of the cohort, and the median age was 79.5 years. Medication lists were available for 36 patients in the EMR; among these, 27 (75%) were exposed to extensive polypharmacy (≥10 medications). For four patients, medication lists were not recorded in the EMR, as they were only seen in the emergency department and were not admitted. Patient characteristics are presented in Table 1. Seven patients could not be followed up due to lack of access to EMR data from primary care after discharge, resulting in 48 recommendations to assess.

### 3.2. Clinical Relevance

**Figure 2:**
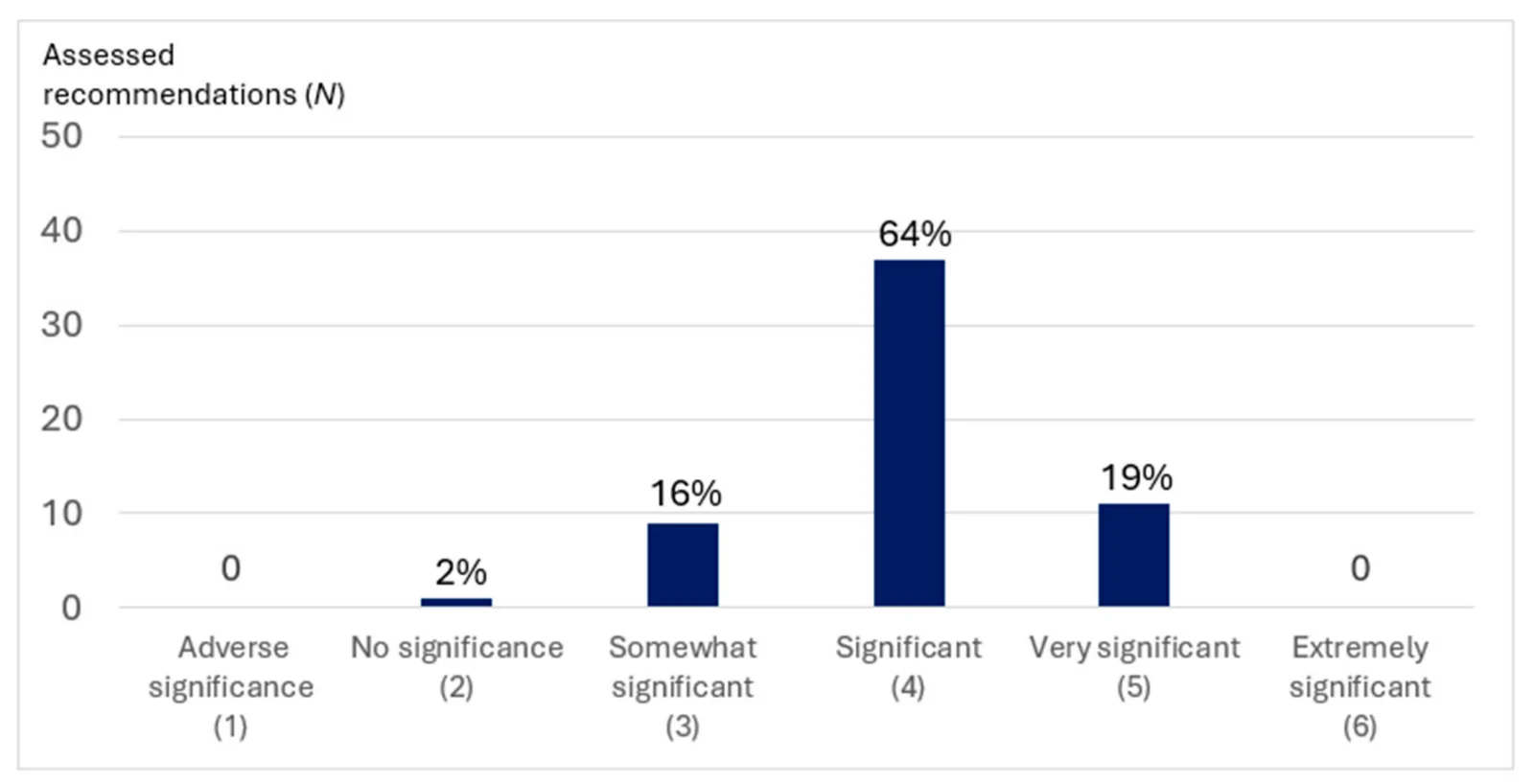
Clinical relevance of recommendations (*N* = 58), according to Hatoum classification scale [26].

**Figure 3:**
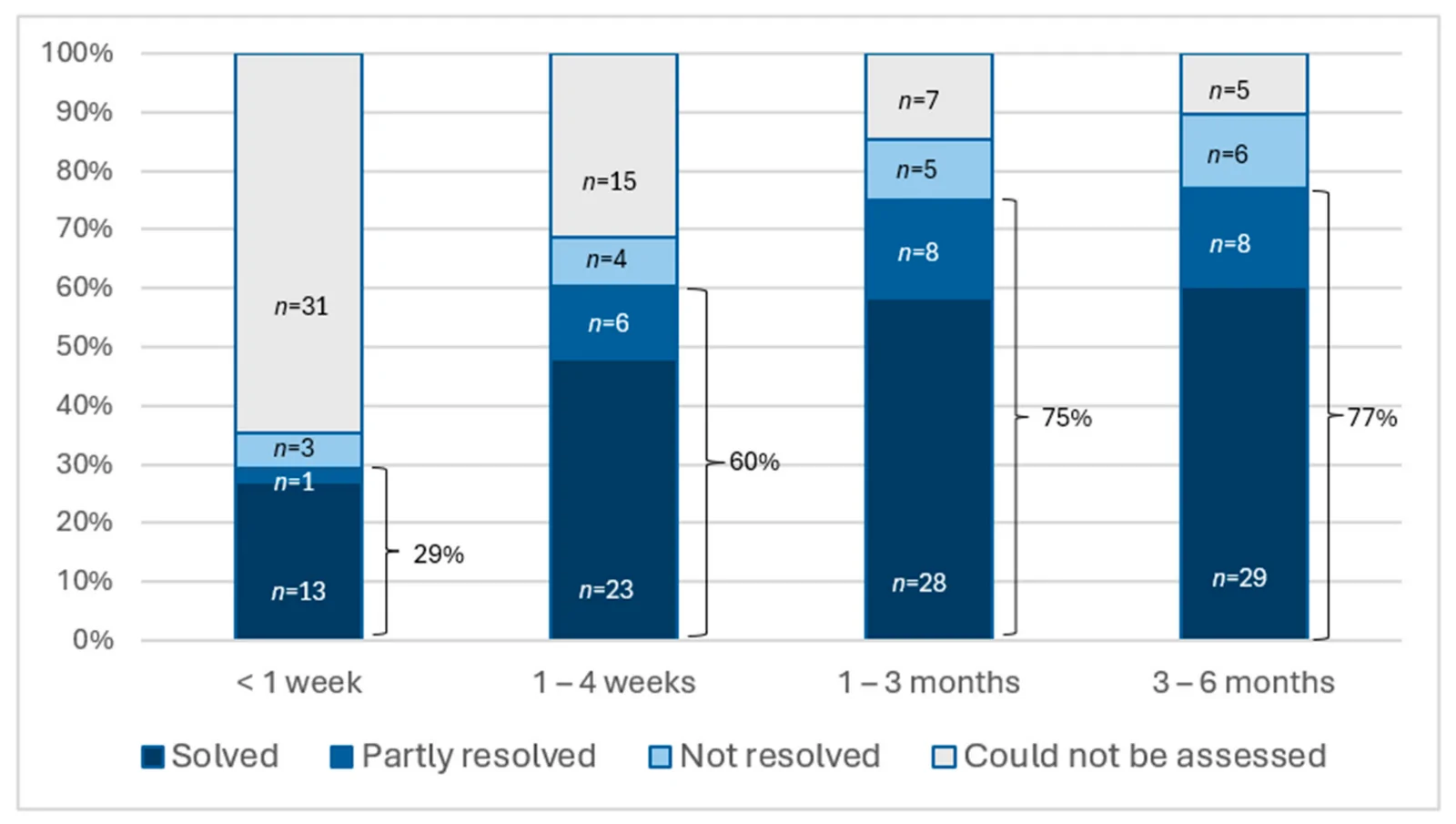
Follow-up on action taken or not for recommendations from MRPs in EMR in primary care (*n* = 48). After 6 months, 77% (*n* = 37) were solved or partly resolved.

In total, 58 MRPs were identified and transferred through 40 referrals initiated by 17 pharmacists. Most referrals contained a single MRP (67.5%), although up to three MRPs were included per referral. The majority of referrals were written by pharmacists (80%). All but one recommendation was rated as at least “somewhat significant” or higher in clinical relevance (Figure 2), and examples of clinically relevant recommendations assessed according to the Hatoum scale are described in Table 2. Inter-rater agreement between the GP and geriatrician was almost perfect (weighted Cohen’s kappa = 0.821). Four of eight physician-written referrals were assessed as lower quality due to missing information from interprofessional medication reviews. Follow-up data were available for 33 referrals covering 48 MRPs; seven referrals (10 MRPs) could not be assessed due to missing EMR access. Among evaluated cases, 77% of MRPs were either resolved (*n* = 29) or partly resolved (*n* = 8) (Figure 3). No statistical association between clinical relevance and acceptance could be detected (odds ratio 2.27, *p* = 0.247, CI 0.57–9.04).

### 3.3. Description of MRPs

**Figure 4:**
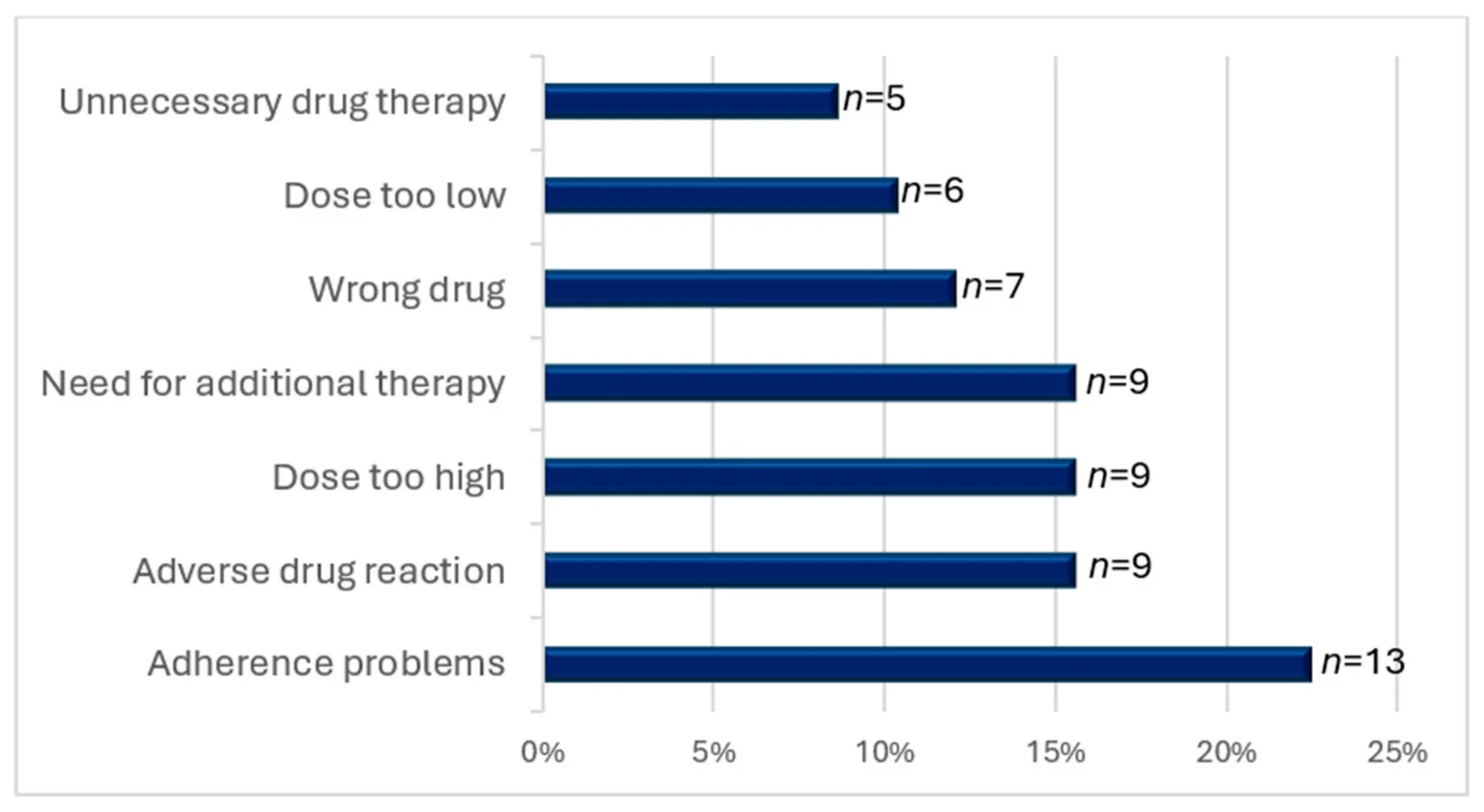
Distribution of MRPs (*N* = 58) in seven categories according to Strand et al. [25].

MRPs were distributed across several categories, with *adherence problems* being the most common (22%) (Figure 4). No statistically significant difference in adherence-related MRPs was observed between patients with and without multidose drug dispensing (*p* = 0.624). Most adherence problems involved cardiovascular medications. Other frequent MRPs included *adverse drug reactions* (16%), *dose too high* (16%), and *need for additional therapy* (16%). Multiple medications could be associated with each MRP. Among MRPs related to *adverse drug reactions* and *dose too high*, 9 of 18 involved medications were in the nervous system group. Overall, medications acting on the nervous system were most frequently involved (23%), including antidepressants, analgesics, and sedatives such as selective serotonin reuptake inhibitors, opioids, and propiomazine. Medications affecting the alimentary tract and metabolism accounted for 21% of MRPs, including antidiabetics and calcium supplements, followed by medications categorised as cardiovascular system (20%). Antidiabetic drugs were mainly associated with the need for additional therapy, while calcium supplements contributed to the reduced absorption of other medications. MRPs involving cardiovascular drugs were primarily related to *adherence problems* and *adverse drug reactions*, including electrolyte disturbances and physical symptoms.

## 4. Discussion

This study described pharmacist-initiated referrals from hospital care to primary care as part of a newly implemented clinical procedure. Overall, most MRPs identified were clinically relevant, and GPs implemented a large proportion of the recommendations. These findings suggest that the referral process supports medication safety during transitions between hospital and primary care, particularly if further integrated into routine practice.

In the present study, pharmacist-initiated referrals may be viewed as a complementary strategy to facilitate prioritisation of medication changes, reduce unnecessary simultaneous adjustments, and support follow-up of unresolved MRPs in primary care. Healthcare systems operate under constrained resources, and hospital admissions are resource-intensive. Efficient use of resources is therefore essential. Clinical pharmacists contribute to medication safety through the identification and management of MRPs, and greater integration of their expertise may be valuable in transitional care. However, pharmacotherapy management during hospitalisation is complex. Multiple simultaneous medication changes may complicate assessment of treatment effects and adverse drug reactions [2]. In selected cases, postponing medication changes until after recovery from acute illness may be appropriate [29]. Transition of care is a recognised high-risk period for medication errors and communication failures [4,5]. Structured referral systems have been proposed to improve continuity between care settings [6]. Previous research also shows that extensive medication changes during hospitalisation may increase the risk of preventable readmissions [30], thus highlighting the importance of clear prioritisation and communication of post-discharge plans. Evidence supports pharmacist involvement in the discharge processes. A Danish study by Ravn-Nielsen et al. showed that pharmacist participation in medication review, discharge planning, and counselling reduced emergency visits [31].

A key finding was the high implementation rate of recommendations (77%), exceeding the rates reported in previous studies (64–71%) [31,32,33]. Prior studies suggest that oral communication increases the acceptance rate [33], while telephone communication is effective but resource-intensive [32]. In the present study, a high acceptance rate was achieved without verbal communication, as referrals were transmitted as structured written documentation in the EMR. However, comparisons with previous studies should be interpreted with caution because of differences in study design, patient population, healthcare setting, and the nature of interventions. In particular, pharmacists in this study prioritised selected MRPs for referral, which may have influenced acceptance rates. This study suggests that well-designed written referral systems are effective means of communicating recommendations when integrated into clinical workflows, particularly where time constraints limit direct communication, and pharmacists are not routinely involved in discharge processes. The structured format may also contribute to clearer allocation of responsibility and facilitate clinical action. Trust in pharmacists’ competence may also explain the high acceptance rates. Studies from Sweden, Canada, and the UK have reported strong pharmacist trust of GPs [15,32,34,35]. There were no recommendations assessed as significantly adverse or extremely significant, which may contribute to the credibility of the recommendations. We did not expect any recommendations to be extremely significant since those should have been resolved and prioritised during the hospital stay. Strong inter-rater agreement between the GP and geriatrician supports the reliability of the clinical relevance assessment and aligns with previous studies showing substantial agreement between professionals [36]. Both assessors being physicians strengthens validity, as GPs are responsible for final medical clinical decisions.

The relatively low number of referrals likely reflects suboptimal implementation. Nevertheless, pharmacists identified clinically relevant MRPs suitable for referral, indicating clinical usefulness. Our previous qualitative research identified barriers such as limited continuity and lack of routine pharmacist involvement as well as facilitators such as organisational support and team integration [37]. These factors are important for future implementation. A small proportion of referrals were written by physicians and more often contained incomplete medication information, suggesting reduced quality when pharmacist involvement is absent. These findings suggest that pharmacist participation contributes to greater consistency and completeness.

*Adherence problems* was the most frequent MRP category, consistent with evidence that non-adherence is a major cause of MRPs and hospital admissions [38]. Patient engagement is essential to improve adherence, especially in older patients with polypharmacy [2]. This highlights the need for structured follow-up across transitions. Pharmacists are trained to discuss medication issues with patients and could take on an important role in medication management and at discharge. Internationally, pharmacists are increasingly integrated into discharge processes in Australia, the United States (US), and the UK, contributing to medication reconciliation and counselling [38,39,40,41,42,43]. This supports expanding pharmacist roles in transitional care.

Other MRPs included *adverse drug reactions*, *dose too high*, and *need for additional therapy*. *Adverse drug reactions* are difficult to assess during acute illness and require follow-up. Pharmacists may identify more adverse drug reactions than other professionals [44], highlighting their role in medication safety. MRPs such as *dose too high* and *need for additional therapy* reflect opportunities for optimisation in older patients with polypharmacy. The population was predominantly older, which is consistent with previous studies [31,33,45]. Regardless of MR -category, the ATC groups most frequently associated with MRPs were the nervous system, cardiovascular system, and alimentary tract and metabolism groups, reflecting their high prescribing prevalence and their vulnerability to MRPs.

### 4.1. Strengths and Limitations

One strength of this study is that it represents one of the first Swedish evaluations of a structured pharmacist referral process and includes multiple hospitals across a range of hospital wards and medical specialties. This increases the transferability of the findings and indicates that the approach may be feasible to implement within existing discharge processes across different healthcare settings. Another strength is that this study includes the entire procedure from the identification and classification of MRPs at hospital, assessment of the clinical relevance of recommendations, to implementation by GPs in primary healthcare. This approach enables a more comprehensive assessment of whether pharmacist recommendations result in implementation during transitions of care and provides insights into their potential contributions to medication management.

An additional strength is the independent and multiprofessional assessment of the clinical relevance of recommendations through the involvement of both a geriatrician and a GP. Since the study population mainly consisted of older patients with extensive medication use, the combination of hospital and primary care perspectives was particularly relevant. The high implementation rate of recommendations in primary care may also support the assessment of the recommendations’ relevance as clinically applicable.

A limitation of this study is the small number of included patients and referrals, meaning that the results should be interpreted as exploratory and primarily used for hypothesis generation rather than for drawing definitive conclusions. The low number of referrals, together with the large variability in the data, may have limited the ability to achieve statistical significance. Furthermore, the low number of referrals may have been due to the early stage of implementation, pharmacists prioritising the most clinically relevant cases, or practical barriers to fully using the referral process. The fact that approximately half of the pharmacists used the procedure indicates that the approach is feasible but that further support and implementation strategies may be required to achieve more consistent use.

Another limitation of this study is the lack of a control group, which made it difficult to determine whether the observed findings were specifically attributable to the pharmacist referrals. Instead, the pragmatic approach included MRPs identified by pharmacists where referrals were initiated. As the information provided in the discharge summary did not explicitly prompt further action, it was unlikely to have facilitated follow-up in primary care. This study reflects real-world clinical practice, which also introduced variation in the structure and content of referrals, which may have affected consistency and limits the conclusions that can be drawn regarding the effect of the referral process itself.

Selection bias is another possible limitation, as patients with cognitive impairment were excluded because informed consent could not be obtained. This may affect generalisability, since this patient group is often targeted for medication reviews by clinical pharmacists. We also lacked information about how many and if any patients declined study participation. However, this study included older patients with polypharmacy, which is the patient group commonly targeted by clinical pharmacists and where the risk of MRPs is particularly relevant. There is no reason to believe that patients with cognitive impairment would benefit less from a referral. However, a more representative sample could have provided a broader understanding of the prevalence and types of MRPs.

Finally, the lack of complete follow-up data from some EMRs is a limitation. This may have affected the ability to assess the outcomes of certain recommendations. However, as the follow-up included both public and private primary care centres and no systematic differences were expected between these groups, the risk of systematic bias due to missing data is considered limited.

### 4.2. Future Research

Future studies on how to improve and implement pharmaceutical interventions concerning transition of care are needed to improve patient safety. The aspect of the GPs’ and patients’ perspectives could add further knowledge to improve the process.

## 5. Conclusions

The referrals initiated by clinical pharmacists were generated to a limited extent. Nevertheless, the results suggest that further implementation of the process is warranted, as the recommendations were of high clinical relevance as were reinforced by the high proportion of actions on MRPs taken by GPs.

## Figures and Tables

**Table 1:** Baseline characteristics of participants in analysis of referrals initiated by clinical pharmacists (*N* = 40). Continuous variables are presented as median (interquartile range, IQR), and categorical variables as number and percentage.

Characteristics	
Female, ***n*** (%)	28 (70)
Age, median, years (IQR)	79.5 (74.0–83.5)
Renal function, median, estimated glomerular filtration rate, mL/min (IQR)	65 (57–79)
Total number of medications *, median, ***n*** (IQR)	11 (9.5–17.5)
Number of continuous medications * median, ***n*** (IQR)	8.5 (6.5–12.5)
Number of mediations when needed * median, ***n*** (IQR)	3 (2.0–5.0)
Service, multidose dispensing of medication, ***n*** (%)	9 (23)
Number of days admitted to hospital, median, ***n*** (IQR)	6.5 (4.5–10.5)
Ward specialty	
Medicine departments (medicine, neurology, infectious diseases) ***n*** (%)	23 (57.5)
Surgery departments (general surgery, orthopaedic) ***n*** (%)	12 (30)
Outpatient clinics (emergency department, psychiatry department) ***n*** (%)	5 (12.5)

* Data for 36 participants analysed as outpatients at emergency departments lack medication lists in EMRs.

**Table 2:** Description of ranking steps of clinical relevance of recommendations according to Hatoum et al. [26].

Ranking	Label	Interpretation	Examples of Assessed Recommendations
1	Adverse significance	Recommendation supplied may lead to an adverse outcome	- *
2	No significance	Recommendation is informational (not specifically related or meaningful to the patient in question)	During medication reconciliation at xx, I noted that the patient is currently not using semaglutide injection. The medication was last dispensed on xx (approximately two years ago). During our conversation, the patient stated that she would like to restart the treatment. The patient is aware that treatment for weight loss is not covered by the pharmaceutical benefits scheme and is willing to pay for the treatment herself. I would be grateful for your assessment regarding whether semaglutide may be reinitiated.
3	Somewhat significant	Benefit of the recommendation to the patient could be neutral depending on professional interpretation.	During a medication review, it was noted that … The patient has also been treated with citalopram 30 mg once daily since nearly 10 years, according to the medical record. I would appreciate your consideration of the ongoing need for citalopram therapy, including whether dose reduction or gradual discontinuation may be appropriate, as the patient is unsure whether the treatment is having a beneficial effect.
4	Significant	Recommendation would bring care to a more acceptable and appropriate level.	Interaction associated with impaired absorption of iron and levothyroxine was noted. It is recommended that administration of iron, levothyroxine, and calcium should be separated by at least 2–4 h to ensure optimal absorption. Kindly consider follow-up of the treatment effects of levothyroxine and iron, as well as reassessment of the ongoing need for iron supplementation and calcium therapy.
5	Very significant	Recommendation qualified by a potential or existing major organ dysfunction.	During the medication review, it emerged that the patient has not been taking his medications regularly. According to the prescription monitoring record, the patient has collected his medications almost a year ago. The patient states that he has a large supply of medications at home and finds managing multiple containers and packages burdensome. He would like to receive his medications in multidose-dispensing packaging, as he believes this would make managing his medication regimen easier.
6	Extremely significant	Information qualified by a life or death situation.	- *

* No recommendations were assessed with this Hatoum ranking criterion.

## Data Availability

The data presented in this study are available on request from the corresponding author (K.W.) due to ethical reasons.

## Abbreviations

The following abbreviations are used in this manuscript:

ATC	Anatomical Therapeutic Chemical
DMS	Discharge Medicines Service
EMR	Electronic medical record
GP	General practitioner
MRP	Medication-related problem
UK	United Kingdom
US	United States of America
